# Practical Manual of the Diseases of the Heart and the Great Vessels

**Published:** 1843-10-14

**Authors:** 


*Practical Manual of the Diseases of the Heart and the Great Vessels; a Work intended to Facilitate and Extend the Study of these Diseases.* By F. A. Aran, Interne of the first class of the Hotel Dieu, &c. &c. *Translated from the French.* By William A. Harris, M. D. Philadelphia: Ed. Barrington & Geo. D. Haswell. 1843. 8vo. pp. 164.

This work, though issued under the modest title of a manual, is yet so comprehensive as to afford minute instruction on every point of which it treats. The author has condensed within a narrow compass every thing valuable from the treatises of Senac, Corvisart, Laennec, Bertin, Bouillaud, Gendrin, and more especially from the work of the late Doctor Hope of London.

The arrangement of the various subjects treated of is unexceptionable, and is as follows:

I. The Anatomy and Physiology of the Heart.

II. The Pathology of the Heart.

III. The Inflammatory Diseases of the Heart and large vessels.

IV. Organic Diseases of the Heart and large vessels.

V. Nervous Diseases of the Heart.

There is also an appendix, which embraces hydro-pericardium, herno-pericardium, pneumo-pericardium, and polypi, or sanguineous concretions of the heart.

All Dr. Aran's views are happily and fully illustrated. His instructions regarding the best method of investigating diseases of the heart, are so clear as to be easily comprehended by the dullest capacity. We know of no work better calculated to facilitate and extend the study of cardiac diseases.

The translation by Dr. William A. Harris, a highly intelligent and promising young physician of this city, is very faithfully and cleverly done. We hope that the success of the present undertaking will induce him to give us further contributions of the same kind.

